# From Rare to Common: Genetic Insights into *TLR7* Variants in a Multicentric Spanish Study on COVID-19 Severity

**DOI:** 10.1007/s10875-025-01892-0

**Published:** 2025-05-27

**Authors:** Arnau Antolí, Gardenia Vargas-Parra, Angels Sierra-Fortuny, Jose Luis Gomez-Vazquez, Paula Rofes, Elisabet Munté, Julen Viana-Errasti, Raúl Marín-Montes, Adriana López-Doriga, Lidia Feliubadaló, Jesús del Valle, Alexandre Pérez-González, Eva Poveda, Xavier Solanich, Conxi Lázaro

**Affiliations:** 1 Internal Medicine Department, Bellvitge University Hospital, L’Hospitalet de Llobregat, Barcelona, Spain; 2 Adult Primary Immunodeficiency Unit (UFIPA), Bellvitge University Hospital, L’Hospitalet de Llobregat, Barcelona, Spain; 3 The Systemic, Vascular Diseases and Ageing Group. Bellvitge Biomedical Research Institute (IDIBELL), L’Hospitalet de Llobregat, Barcelona, Spain; 4 Clinical Sciences Department, Faculty of Medicine and Health Sciences, University of Barcelona, Barcelona, Spain; 5 Hereditary Cancer Program, Catalan Institute of Oncology, L’Hospitalet de Llobregat, Barcelona, Spain; 6 Molecular Mechanisms and Experimental Therapy in Oncology Program, Bellvitge Biomedical Research Institute (IDIBELL), L’Hospitalet de Llobregat, Barcelona, Spain; 7 Centro de Investigación Biomédica en Red de Cáncer (CIBERONC), Instituto de Salud Carlos III, Madrid, Spain; 8 Unit of Bioinformatics for Precision Oncology, Catalan Institute of Oncology, L’Hospitalet de Llobregat, Barcelona, Spain; 9 Nennisiwok AI Lab, Barcelona, Spain; 10 Internal Medicine Department, Complexo Hospitalario Universitario de Vigo (CHUVI), SERGAS, Vigo, Pontevedra, Spain; 11 Virology and Pathogenesis, Galicia Sur Health Research Institute (IIS Galicia Sur), SERGAS-UVIGO, Vigo, Pontevedra, Spain

**Keywords:** TLR7, X-linked, COVID-19, immunodeficiency, innate immunity

## Abstract

*TLR7*, which encodes a key receptor for single-stranded RNA (ssRNA) virus of the innate immune system, was recently associated with X-linked immunodeficiency and COVID-19 susceptibility. This study investigates the association between *TLR7* variants and susceptibility to severe COVID-19 in a multicentric Spanish cohort. The *TLR7* gene was sequenced in a cohort of 365 COVID-19 patients, stratified into two groups: one comprising mild and asymptomatic patients, considered as controls (*n* = 87), and the other consisting of moderate to severely affected patients hospitalized due to COVID-19 pneumonia, considered as cases (*n* = 278). A total of 152 unique *TLR7* variants were identified, of note, six rare variants were identified in 11 cases (3.96%), all of whom belonged to the case group. The functional impact of rare *TLR7* variants was assessed using a luciferase reporter assay and revealed that N215S is a loss-of-function (LOF) variant, while D332G exhibits an hypomorphic behavior. Conversely, H90Y, V219I, A448V, and R902K maintained normal signaling. No skewed X-inactivation was observed in female carriers of N215S or D332G. In addition, the common variants Q11L (rs179008), c.4-151A>G (rs179009) and c.*881C>G (rs3853839) were associated with severe pneumonia, while c.4-151A>G (rs179009) was specifically linked to Intensive Care Unit (ICU) admission. These findings highlight the role of TLR7 in antiviral immune response and its association with severe COVID-19 in men. The luciferase assay proves to be a reliable tool for evaluating TLR7 signaling, effectively distinguishing between neutral, LOF, and gain-of-function (GOF) variants. Further research is needed to better understand *TLR7* variants and its implications in immunodeficiency and immune dysregulation.

**Supplementary Information:**

The online version contains supplementary material available at 10.1007/s10875-025-01892-0.

## Introduction

Located on the X chromosome (Xp.22.2), the *TLR7* gene was described in 2000 [1, 2]. *TLR7* comprises 3 exons, with exon 2 encoding only the initiation methionine, while the remainder part of the protein is encoded in exon 3 [1–3]. TLR7 is a pattern recognition receptor that feature an extracellular leucine reach repeats (LRR) domain and a cytoplasmic Toll/IL-1 domain [4]. TLR7 is expressed in the endosomal compartment of plasmocytoid dendritic cells (pDCs) [5]. Its known ligands include imidazoquinolinone derivatives (IMQs), such as imiquimod (R837), ssRNA viruses, and synthetic uridine-rich ssRNA sequences that mimic viral RNA. TLR7 contains two distinct ligand-binding sites: site 1, which recognizes nucleosides, nucleoside analogues and IMQs, playing a key role in receptor dimerization; and site 2, which binds short oligoribonucleotides and enhances the binding affinity of site 1 ligands to facilitate dimerization. Upon activation, the LRR domain forms an M-shaped dimer containing the two ligand-binding sites [6]. TLR7 signaling is mediated through a MyD88-dependent pathway that activates anti-viral immune response. This pathway recruits the IRF7 signaling mediator and, through the adaptor molecule TRAF6, activates the transcription factor NF-κB, resulting in a substantial secretion of interferon (IFN)-α in pDCs [5–11].

No genetic defects or clinical conditions were associated with *TLR7*, until 2020, when Van der Made et al. reported two pairs of unrelated siblings with severe COVID-19 carrying pathogenic *TLR7* variants [12]. The SARS-CoV-2 pandemic, resulting in over 760 million cumulative cases and nearly seven million deaths worldwide [13–15] allowed the identification of advanced age as the most significant common risk factor for severe disease [16–19]. Other underlying medical conditions, such as chronic lung disease and diabetes, also increased the susceptibility to severe COVID-19[20]. Notwithstanding, severe COVID-19 cases were observed in previously healthy young individuals too. Our group reported a rare *TLR7* N215S variant in two healthy brothers who experienced severe COVID-19[21]. Subsequently, the accumulated evidence, led to the description of a new inborn error of immunity known as X-linked *TLR7* deficiency for severe COVID-19[22–24]. The comprehensive study of genetic susceptibility to COVID-19 has provided substantial evidence that rare *TLR7* variants are associated with an increased risk of developing severe forms of the disease [25–29]. In contrast, the association between the common *TRL7* variants and COVID-19 severity has yielded conflicting results [30–34]. Next-Generation Sequencing (NGS) studies performed during the COVID-19 pandemic marked a paradigm shift, revealing genetic factors influencing SARS-CoV-2 susceptibility [12, 21, 22, 29, 35–39]. Proper TLR7-mediated viral sensing and Myddosome signal transduction are crucial for an early and robust type I interferon (IFN-I) response, ensuring effective viral control and mild or asymptomatic disease. Conversely, delayed IFN-I induction or dysregulated responses may lead to excessive TLR7 protein levels or mislocalization, triggering inflammasome activation and cytokine storm development [32, 40–42].

In this context, our study aims to evaluate the contribution of rare and common *TLR7* variants to COVID-19 severity in a multicenter Spanish cohort, including the functional analysis of selected rare variants.

## Methods

### Study Design

*TLR7* variants were screened in a cohort of SARS-CoV-2 primary infected patients. The complete clinical cohort comprises 365 COVID-19 patients from two hospitals: Bellvitge University Hospital, Barcelona, and Instituto de Investigación Sanitaria Galicia Sur (IISGS), Pontevedra, both located in Spain. Samples from the IISGS were selected from individuals who belonged to the COVID cohort. For the current study, cases (*n* = 278) were defined as patients developing COVID-19 pneumonia with a World Health Organization (WHO) Ordinal Scale (WHO-OS; Table S1) of ≥ 3; Controls (*n* = 87) were defined as WHO-OS ≤ 2. All collected demographic and clinical data are shown in Table S5.

### DNA Extraction

DNA was isolated from buffy coat samples using a Maxwell® 16 Instrument and Maxwell Blood DNA purification kit (AS1010, Promega, Madison, WI, USA), following the manufacturer's protocol.

### Genetic Testing

Genetic testing was performed on genomic DNA using a NGS custom-designed panel. This panel includes the coding sequence and at least surrounding 20bp for 136 genes and 55 Single Nucleotide Polymorphisms (SNPs) (Table S2) to assess COVID-19 human genetic susceptibility. For the *TLR7* gene, the entire gene sequence was captured, including Untranslated Regions (UTRs), exons and introns, based on the NG_012569.1. Library preparation was performed following KAPA HyperCap Workflow v3.0 (Roche, Basel, Switzerland). Capture enriched libraries were sequenced on a NextSeq 550 instrument, with 2×151 paired-end cycles (Illumina, San Diego, CA, USA). *TLR7* N215S variant proband’s and family members [21] were included in the analysis as part of the quality control for the NGS panel.

### Bioinformatics Analysis

NGS data were processed using a custom bioinformatics pipeline based on standard tools. Raw FASTQ files were processed using fastp [43] with default parameters, and they were aligned against the UCSC GRCh37/hg19 human reference genome using bwa-mem2 [44]. Then, following the GATK Best Practices recommendations [45], duplicate removal, base quality score recalibration and single-sample germline short variant discovery over the target regions were performed using their GATK4 modules [46] with the default parameters. DeCoN V2.0.1 was used for Copy Number Variation (CNV) detection. The obtained Single Nucleotide Variants (SNV)s and insertions and deletions (indels) were specifically hard filtered using standard parameters with VariantFiltration GATK4 module: (i) SNP filters were QD < 2.0, QUAL < 30.0, FS > 60.0, SOR > 3.0, MQ < 40.0, MQRankSum < −12.5 and ReadPosRankSum < −8.0, and (ii) indel filters were QD < 2.0, QUAL < 30.0, FS > 200.0 and ReadPosRankSum < −20.0. Then, PASS variants were normalized and multiallelic sites were split using LeftAlignAndTrimVariants GATK4 module. Finally, variants were annotated integrating VEP [47], ANNOVAR [48] and SnpEff [49] to provide information about the gene locus, functional impact, specific variation databases (dbSNP and ClinVar), population frequencies (1000G, ESP6500, gnomAD and ALFA) and in silico predictors of pathogenicity (SIFT, Polyphen2, CADD, MutationAssessor, REVEL, METALR, MetaLR and MetaRNN). In addition, specific sequencing and alignment quality metrics were generated from FASTQ and BAM files using FastQC [50] and GATK4, respectively, and coverage metrics were obtained using Mosdepth [51]. Then, they were collected and displayed in a report using MultiQC [52].

### Cell Culture

Human embryonic kidney (HEK) 293^T^ cells (CRL-1573, ATCC, Manassas, VA, USA) were grown in Dulbecco’s modified Eagle medium (DMEM) (31,966,021, Thermo Fisher Scientific, Waltham, MA, USA) supplemented with 10% (v/v) fetal bovine serum (A5256701, Thermo Fisher Scientific) and maintained at 37°C with 5% CO2.

### Plasmids

A *TLR7* vector template (pCMV6-TLR7) was generated by inserting *TLR7* (RC207515, OriGene, Rockville, MD, USA) into a pCMV6-AC-Myc-DDK Mammalian Expression Vector (PS100007; OriGene). The In-Fusion® Snap Assembly Value Bundle (638,946, Takara Bio USA, San Jose, CA, USA) was used to generate all the *TLR7* variants according to the manufacturer’s instructions. The primers for site-directed mutagenesis were used according to Asano et al. [22]; those not previously described are listed in Table S3. All variants were subsequently confirmed by Sanger sequencing (Figure S1).

### Western Blot

HEK293^T^ cells were seeded in 6-well plates in 10% FBS-supplemented DMEM. After 24h, cells were transfected with wild-type (WT) or variant *TLR7* vectors in the presence of X-tremeGENE 9 DNA transfection reagent (06365809001, Roche). After 24h, protein extraction was made lysing cells in RIPA buffer supplemented with protease/phosphatase inhibitors (4693116001, Roche). Protein concentration was determined with the Pierce BCA Protein Assay Kit (23225, Thermo Fisher Scientific). Western blot was performed using 20 µg of total protein extract in 12% acrylamide gels (1610185, Bio-Rad, Hercules, CA, USA). Protein transference to nitrocellulose membranes was performed with a Trans-Blot Turbo™ RTA Transfer Kit (170–4270, Bio-Rad) in the Trans-Blot Turbo™ Transfer System (Bio-Rad). Membranes were blocked with 5% BSA (10735078001, Roche) for 1h. Membranes were incubated overnight at 4ºC with primary antibodies diluted 1:1000 for N-terminus TLR7 (5632, Cell Signaling Technology, Danvers, MA, USA) or C-terminus TLR7 (EPR2088(2), Abcam, Cambridge, UK) and 1:2500 GAPDH (ab9485, Abcam). Detection was performed using 1:1000 Goat anti-Rabbit IgG (H+L) secondary antibody (32460, Thermo Fisher Scientific).

### TLR7 Luciferase Reporter Assay

HEK293^T^ cells were seeded in 96-well plates in 10% FBS-supplemented DMEM. After 24h, cells were transfected in the presence of X-tremeGENE 9 DNA transfection reagent with a vector containing five copies of an NF-κB response element, followed by the luciferase reporter gene *luc2P* (E8491, Promega), 100 ng/well; either the WT or the variant pCMV6-TLR7 vector, 20 ng/well; the *UNC93B1* Human Tagged ORF Clone vector (RC210505, OriGene), 0.625 ng/well; and a constitutively expressing *Renilla* luciferase plasmid (E2231, Promega), 10 ng/well. After 24h, cells were stimulated or not with various TLR7 agonists for 24h: R848 (1 µg/ml), R837 (5 µg/ml) or CL264 (5 µg/ml) (tlrl-r848-1, Invivogen, San Diego, CA, USA). Luciferase and *Renilla* activity were measured using the Dual-Luciferase® Reporter Assay System (E1960, Promega). Renilla-Luciferase assay (RLA) ratios were normalized against the stimulated WT values. Those variant ratios showing less than 25% of the activity of the stimulated WT were considered loss-of-function (LOF) [22, 53].

### Skewed X Inactivation Analysis

Skewed X-inactivation was assessed via the HUMARA assay [54]. DNA samples were either digested with HpaII (R0171S, New England Biolabs, Ipswich, MA, USA) or incubated without the enzyme. The androgen receptor locus was then PCR-amplified using FAM-labeled primers [forward FAM-labeled primer (5’-GCTGTGAAGGTTGCTGTTCCTCAT-3’) and a reverse primer [5’-TCCAGAATCTGTTCCAGAGCGTGC-3’] and analyzed on an AB3500 instrument (Applied Biosystems).

### Statistical Analysis

For statistical analysis of common *TLR7* variants, group comparisons were performed using the chi-square test. Statistical significance was set at p < 0.05, and odds ratios (OR) with 95% confidence intervals (CI) were calculated. Analyses were conducted using SPSS, version 19.

## Results

### *TLR7*  Variants

This study identified 152 unique *TLR7* variants in 365 patients (Table S4). Of them, 126 were SNVs and 26 were small insertions and deletions (indels). Among SNV variants, 114 were intronic and 12 were exonic. Amid exonic variants, five were synonymous and seven missense. All indels were deep intronic and no canonical splice site, nonsense, frameshift variants or CNVs were identified in our cohort. The common missense variant Q11L was found in 106 patients in our cohort, representing an allele frequency of 0.29. All rare missense variants [minor allele frequency (MAF): < 0.01] were considered for further study: V219I, A448V, R920K, D332G, N215S and H90Y. Since in silico tools did not predict any deleterious splicing effects, synonymous and intronic variants were excluded from functional validation.

### *TLR7 *Rare Variant Carriers

The six rare *TLR7* missense variants were identified in eleven patients, all classified as cases, hospitalized due to COVID-19 pneumonia. These variants accounted for 3.01% of the cohort and had a joint prevalence of 3.96% among cases (Table 1). No rare *TLR7* variants were found in the control group.

**Table 1:** Rare *TLR7* variant carriers in our cohort

Gene	HGVSc	HGVSp	Variant	AFS	MAF	References	Effect*	Patient	Sex	Age	Ethnicity	WHO-OS	Allele status
*TLR7*	c.268C>T	p.His90Tyr	H90Y	0.00274	-	-	Neutral	231	Woman	69	European	7	Heterozygous
	c.644A>G	p.Asn215Ser	N215S	0.00274	-	21	LOF	1	Man	30	Latino	5	Hemizygous
	c.655G>A	p.Val219Ile	V219I	0.013698	1.27e-3	22,29	Neutral	4	Man	48	Latino	4	Hemizygous
								70	Man	31	Latino	5	Hemizygous
								100	Man	49	Latino	7	Hemizygous
								165	Man	46	Latino	8	Hemizygous
								262	Man	44	Latino	6	Hemizygous
	c.995A>G	p.Asp332Gly	D332G	0.00274	7.44e-6	22	Hypomorphic	148	Woman	48	European	5	Heterozygous
	c.1343C >T	p.Ala448Val	A448V	0.0055	4.16e-3	22,29	Neutral	11	Man	31	European	3	Hemizygous
								116	Man	58	European	7	Hemizygous
	c.2759G>A	p.Arg920Lys	R920K	0.00274	1.19e-4	22,29,37	Neutral	204	Woman	65	European	7	Heterozygous

*AFS* allele frequency among the samples, *MAF* minor variant allele frequency according to gnomAD v4, *LOF* Loss of function, *WHO-OS* WHO Ordinal Scale. *According the TLR7 luciferase reporter assay

H90Y was identified in heterozygosity in a 69-year-old woman of European ancestry, who had no known risk factors for severe COVID-19 disease other than her age. She contracted COVID-19 and experienced a critical course, reaching grade 7 in the WHO-OS, requiring oral intubation, invasive mechanical ventilation (IMV), and posterior tracheostomy due to acute respiratory distress syndrome (ARDS). The H90Y variant was not found in population databases and has not been previously reported in any patient.

The N215S variant was found in hemizygosity in patient 1. The proband, previously described by Solanich et al.[21], is a 30-year-old man of Latino ancestry without risk factors for COVID-19, yet he developed a severe disease. N215S was not present in population databases and was also found in hemizygosity in the proband’s brother and in heterozygosity in his mother. The variant implies a change in a highly conserved nucleotide in the TLR7 LRR domain, and in silico predictors inferred a possibly damaging effect. Unfortunately, functional validation of this variant was not possible at that time [21].

The V219I variant was found in hemizygosity in five unrelated patients. All were men of Latino ancestry, with or without risk factors for severe disease, and were hospitalized due to COVID-19 pneumonia. These patients exhibited a wide range of COVID-19 severity, detailed in Table S5. Three of them developed critical disease with ARDS, one of whom died despite receiving extracorporeal membrane oxygenation (ECMO).

Variant D332G was identified in heterozygosity in a 48-year-old woman with obesity, a known risk factor for severe COVID-19. She presented a severe disease that required intermediate care admission and respiratory support with high-flow nasal cannula (HFNC).

The A448V variant was found in hemizygosity in two men of European ancestry exhibiting a distinct phenotype. Patient 11, a 31-year-old man without risk factors for severe disease, was hospitalized with COVID-19 pneumonia but did not require oxygen support; patient 116, who was 58-year-old with risk factors, developed a severe disease with ARDS requiring IMV and ECMO.

Lastly, we identified the R920K variant in a 65-year-old woman with multiple risk factors, that developed a critical disease with ARDS, requiring IMV and vasoactive support. She experienced several complications: Takotsubo syndrome, atrial fibrillation, bilateral iliac deep vein thrombosis, and bilateral segmentary pulmonary embolism. She also had lower gastroinstestinal bleeding in the context of anticoagulation therapy, as well as bilateral hydropneumothorax as a consequence of bilateral bronco-pleural fistula and multiple superinfections requiring antibiotic treatment.

We did not identify any additional putative pathogenic variant in the remaining 135 genes in either of the carrier patients described above (data not shown; manuscript in preparation). Additionally, none of these patients showed evidence of autoantibodies neutralizing type I IFNs (AAN-IFN-I) (data not shown; manuscript in preparation). This is relevant considering that phenocopies of type I IFN deficiency could potentially explain susceptibility to COVID-19.

### Functional Evaluation of *TLR7*  Variants

**Fig. 1:**
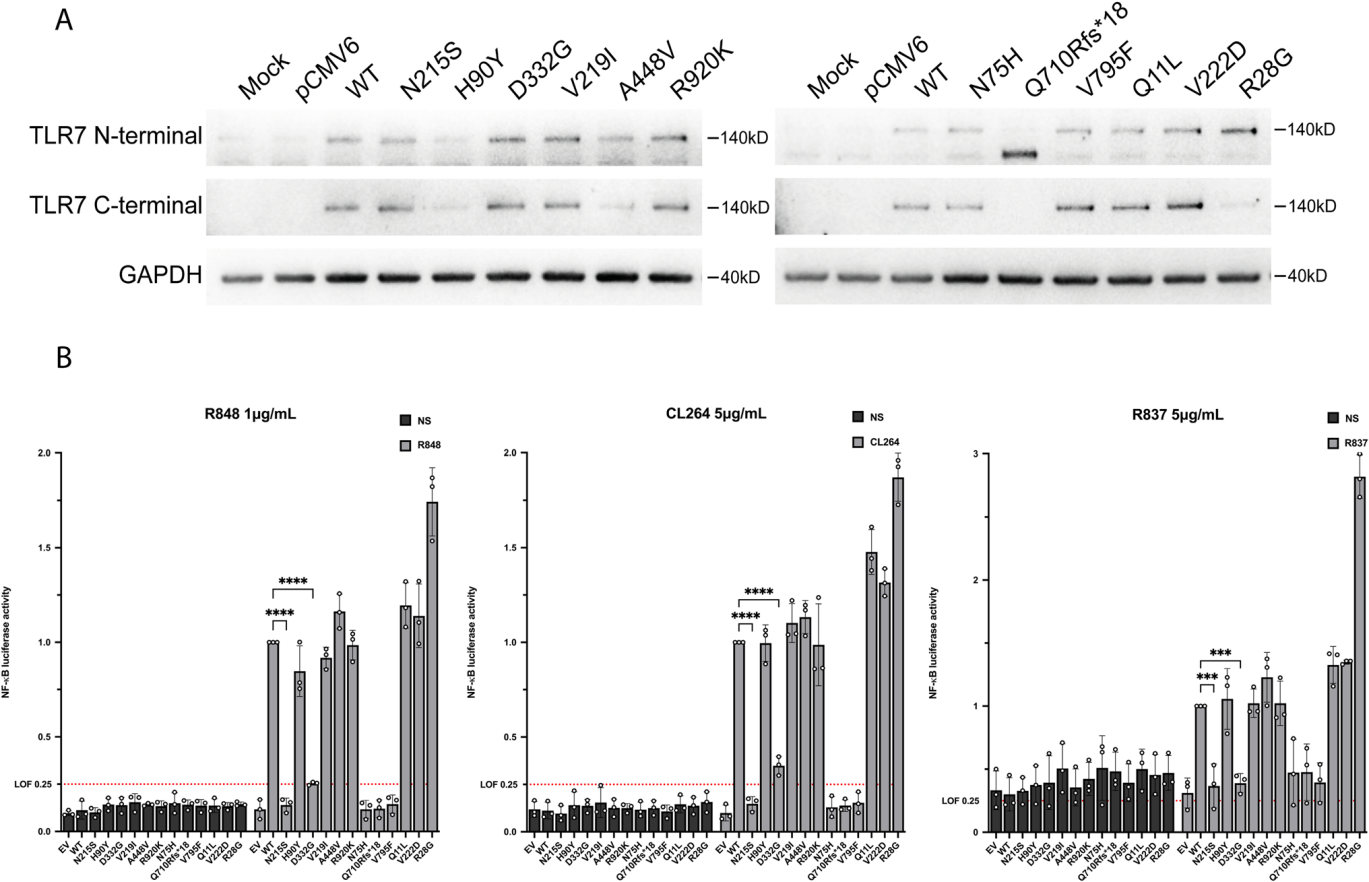
Functional evaluation of the 6 rare *TLR7* variants. Previous known *TLR7* LOF variants N75H, Q710Rfs*18 and V795F (References 12,22), GOF variant: R28G (Reference 71), and common variants Q11L and V222D were included in the functional evaluation as controls. **A** Immunobloting of TLR7 (140kD) WT and variants using N-terminal and C-terminal primary antibodies. **B** HEK293^T^ were or not stimulated with R848 1 μg/mL, CL264 5 μg/mL, R837 5 μg/mL for 24 h. NFκB response was measured using a Dual-Luciferase Reporter, Luciferase/Renilla ratios were normalized against the stimulated WT variant values. Mean ± SEM of *n* = 3 experiments. Two-way ANOVA with Dunnett’s post hoc test. Variants with less than 25% of the activity of the stimulated WT variant were considered LOF. EV: Empty vector; WT: Wild Type; LOF: Loss of function; GOF: Gain of function; NS: non-stimulated; *p < 0.0332; **p < 0.0021; ***p < 0.0002; ****p < 0.0001

To further analyze the putative effect on protein function of the rare variants described above, the six *TLR7* variants were modeled by site-directed mutagenesis and transiently expressed in HEK293^T ^cells, which lack endogenous expression of *TLR7*. As expected, and shown in Fig. 1A, all the *TLR7* variants exhibited normal protein expression except for the frameshift variant Q710Rfs*18, that exhibited a lack of C-terminal immunostaining and a reduced size in the western blot analysis.

The functional evaluation was performed using an in vitro stimulation assay with IMQs (R848, CL264 or R837) and an NF-κB luciferase-reporter assay, according to Asano et al.[22] and C. David et al.[53]. Figure 1B illustrates that the *TLR7* N215S variant exhibited a complete lack of stimulation with IMQs, similar to previously known LOF variants. The unreported variant H90Y exhibited a neutral effect, comparable to the WT after stimulation with the three IMQs, revealing a normal TLR7 function. As previously reported [22], the D332G variant exhibited a lower activity than the WT but consistently above the 25% RLA NF-κB activity, behaving more like a hypomorphic variant than a complete LOF one. Table 1 summarizes the functional results of all rare *TLR7* investigated in the present study.

### Skewed X Inactivation Evaluation in *TLR7* Variant Carriers

We hypothesized that skewed X-chromosome inactivation could be the underlying cause of severe COVID-19 in women carrying N215S (probrand’s mother) and D332G variants. Therefore, we evaluated skewed X inactivation using the HUMARA assay in women carrying these variants (Table 1). The corrected allele ratios of both carriers resulted above 20%, indicating no evidence of skewed X inactivation in these patients (Figure S2).

### Familial Segregation of *TLR7* D332G Variant

**Fig. 2:**
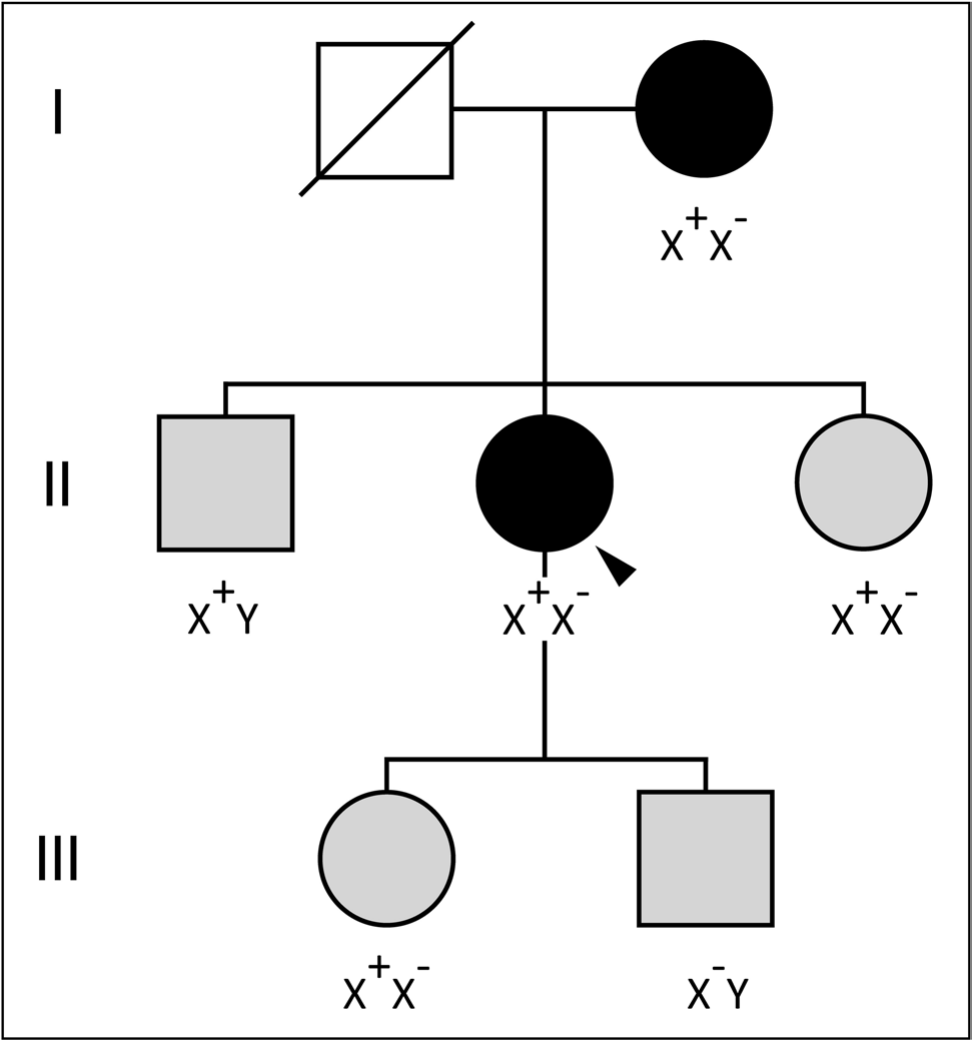
Pedigree of the family harbouring D332G variant. X^+^ indicates D332G allele. X^−^ indicates WT allele. The proband is indicated with an arrow. Black color indicates COVID-19 moderate (WHO-OS 4) to severe phenotype (WHO-OS 5), while grey indicates patients with a mild to asymptomatic (WHO-OS 1–2) disease presentation

The penetrance of *TLR7* hypomorphic defects, such as the D332G variant, remains unknown. Our goal was to establish a genotype–phenotype correlation within the proband’s family, given the hypomorphic nature of the D332G variant (Fig. 2). All women in the family were heterozygous for the *TLR7* D332G variant, while the proband’s brother was the only man presenting the variant in hemizygosity. Interestingly, all family members were simultaneously infected by SARS-CoV-2, but the severity of their disease varied widely. The proband experienced the most severe disease, requiring HFNC and intermediate care admission (WHO-OS 5). The proband’s mother required hospitalization and oxygen therapy (WHO-OS 4). The proband’s sister developed symptoms of COVID-19 pneumonia, including dyspnea, chest pain, and fever; however, she was managed on an outpatient basis, as her initial chest radiograph showed no pulmonary infiltrates, and her oxygen saturation levels were normal. The proband’s brother, his son and daughter all had mild to asymptomatic disease.

### Common *TLR7* Polymorphisms

As TLR7 plays a central role in SARS-CoV-2 detection and the initiation of the innate immune response, several studies have investigated the association between common *TLR7* variants and COVID-19. In Table 2 and Table S6, we analyzed the correlation of these variants and COVID-19 adverse outcomes. A potential effect was observed for variants Q11L, c.4-151A>G and c.*881C>G in COVID-19 cases compared to controls, pneumonia requiring supplemental oxygen with a fraction of inspired oxygen (FiO2) greater than 31% and WHO-OS score ≥ 5. Notably, the intronic variant c.4-151A>G was the only variant related to ICU admission due to COVID-19, with an OR of 1.93 (CI 95% 1.13 to 3.32).

**Table 2:** Common *TLR7* variants analyzed in this study and distribution according to different clinical outcomes

*TLR7* SNP (rs from dbSNP)	Genotype	Clinical Outcome				OR (95% CI)	*p*-value
		N	%	N	%		
		Cases (*n* = 278)		Controls (*n* = 87)			
Q11L (rs179008)	T/T—T	55	87.3	8	12.7	**2.436** (1.111 to 5.339)	**0.023**
	T/A—A/A—A	223	73.8	79	26.2		
c.4-151A>G (rs179009)	G/G—G	65	89	8	11	**3.013** (1.384 to 6.564)	**0.003**
	A/A—G/A—A	213	72.9	79	27.1		
c.*881C>G (rs3853839)	G/G—G	60	90.9	6	9.1	**3.7156** (1.5456 to 8.9322)	**0.0034**
	C/C—G/C—C	218	72.9	81	27.1		
		**Pneumonia requiring supplemental oxygen FiO2 ≥ 31%**					
		**Yes (*n* = 275)**		**No (*n* = 90)**			
Q11L (rs179008)	T/T—T	55	87.3	8	12.7	**2.563** (1.170 to 5.611)	**0.016**
	T/A—A/A—A	220	72.8	82	27.2		
c.4-151A>G (rs179009)	G/G—G	65	89	8	11	**3.173** (1.4580 to 6.9034)	**0.002**
	A/A—G/A—A	210	71.9	82	28.1		
c.*881C>G (rs3853839)	G/G—G	59	89.4	7	10.6	**3.2388** (1.4216 to 7.3786)	**0.0052**
	C/C—G/C—C	216	72.2	83	27.8		
		**WHO-OS ≥ 5**					
		**Yes (*n* = 266)**		**No (*n* = 99)**			
Q11L (rs179008)	T/T—T	53	84.1	10	15.9	**2.215** (1.078 to 4.548)	**0.029**
	T/A—A/A—A	213	70.5	89	29.5		
c.4-151A>G (rs179009)	G/G—G	63	86.3	10	13.7	**2.762** (1.355 to 5.630)	**0.003**
	A/A—G/A—A	203	69.5	89	30.5		
c.*881C>G (rs3853839)	G/G—G	59	89.4	7	10.6	**3.7460** (1.6480 to 8.5148)	**0.0016**
	C/C—G/C—C	207	69.2	92	30.8		
		**ICU Admission**					
		**Yes (*n* = 199)**		**No (*n* = 166)**			
Q11L (rs179008)	T/T—T	41	65.1	22	34.9	1.699 (0.965 to 2.989)	0.71
	T/A—A/A—A	158	52.3	144	47.7		
c.4-151A>G (rs179009)	G/G—G	49	67.1	24	32.9	**1.933** (1.127 to 3.315)	**0.018**
	A/A—G/A—A	150	51.4	142	48.6		
c.*881C>G (rs3853839)	G/G—G	37	56.1	29	43.9	1.079 (0.631 to 1.845)	0.891
	C/C—G/C—C	162	54.2	137	45.8		
		**Death**					
		**Yes (*n* = 14)**		**No (*n* = 351)**			
Q11L (rs179008)	T/T—T	3	4.8	60	95.2	1.323 (0.358 to 4.885)	0.717
	T/A—A/A—A	11	3.6	291	96.4		
c.4-151A>G (rs179009)	G/G—G	4	5.5	69	94.5	1.635 (0.498 to 5.368)	0.492
	A/A—G/A—A	10	3.4	282	96.6		
c.*881C>G (rs3853839)	G/G—G	3	4.5	63	95.5	1.247 (0.338 to 4.599)	0.725
	C/C—G/C—C	11	3.7	288	96.3		

*SNP* single-nucleotide polymorphism, *FiO2* Fraction of inspired oxygen, *WHO-OS* WHO Ordinal Scale, *ICU* Intensive Care Unit

## Discussion

The COVID-19 pandemic spurred unprecedented biomedical research to address the health crisis. Significant focus was directed toward host susceptibility. These findings established *TLR7* as a cornerstone of the innate immune response to SARS-CoV-2. Here we present the results of an in-depth analysis of *TLR7* variants identified in a Spanish multicenter cohort of COVID-19 patients, finding rare *TLR7* variants in 3.96% unvaccinated hospitalized cases, while no rare variants were detected in controls.

When analyzing *TLR7*, six rare variants were considered relevant for further research. The functional insights confirmed the pathogenicity of the N215S private variant, presenting complete LOF when stimulated with IMQs, reinforcing its deleterious role. We also describe a family carrying the hypomorphic variant D332G. This very rare variant was described previously as hypomorphic by Asano et al. Subsequently, an association study postulated that D332G appeared to be overrepresented among Spanish patients [28]. Despite its impact, the residual function of this variant may be sufficient to establish an innate immune response in hemizygous males, like our proband’s brother, who experienced only mild disease despite sharing the proband’s obesity. We identified a new private missense variant, H90Y. Nevertheless, the functional analysis suggested a likely neutral effect.

Evidence on *TLR7* variants with a higher population frequency, such as V219I, A448V and R920K, is inconsistent. Variant V219I was first described as a COVID-19 susceptibility factor by Fallerini et al. [29]. When analyzed in PBMCs and in HEK293^T^ cells, it showed a hypomorphic effect by impairing *IRF7* and *IFNγ* mRNA expression upon stimulation with IMQs. Conversely, Asano et al. [22] found a normal *TLR7* function in a HEK293^T^ functional assay. Thereafter, Mantovani et al. [37] performed an RNA-Seq analysis on PBCMs after stimulation with IMQs and observed impaired upregulation of *IFNγ*. Interestingly, we identified V219I in five hospitalized Latino-ancestry males, with an allelic frequency (AF) of 0.0137 in our cohort, aligning with its frequency in the Latino/Admixed American population (MAF: 0.02602), but remaining globally rare (MAF: 0.00127) according to gnomAD. The A448V variant only presented an impaired *IFNγ* upregulation on PBMCs [37], while the remaining functional studies suggested no significant disruption of TLR7 function [22, 29]. Regarding R920K variant, Mantovanti et al. observed a profound impairment of TLR7 signaling pathway in PBMCs from a patient carrying the R920K variant, with a significant reduction in *IFNα*, *IFNγ*, *RSAD2*, *ACOD1*, and *IFIT2* mRNA levels upon IMQ stimulation [37]. Nevertheless, functional studies performed on patient-derived PBMCs [12, 21, 29, 37] may be influenced by other genetic factors, whereas analyzing isolated variants in an in vitro model with controls allows a more precise assessment [22, 53]. Our results found a functional profile of V219I, A448V and R920K resembling to the WT.

This study highlights that the TLR7 luciferase reporter assay is a reliable and replicable method to evaluate *TLR7* LOF variants. To enhance understanding of this susceptibility, functional validation of these variants is crucial. Chemical ligands, such as IMQs, effectively induce TLR7 dimerization and activation by binding to the first site [6]. Variants that significantly disrupt or enhance TLR7 function could be clearly identified using this method. Nonetheless, TLR7 acts as a dual receptor for guanosine- and uridine-containing ssRNAs [6], and a possible limitation of the TLR7 luciferase reporter assay, as proposed by Asano et al. [22] and David et al. [53], could be the potential underestimation of variants affecting the second site or generating a more physiological defect. TLR7, similar to other endosomal TLRs, is highly conserved and mutation-intolerant, as reflected by its evolutionary constrain [55] and high pLI (probability of being LOF intolerant) score [3]. This highlights the importance of functional validation of all rare variants, even those predicted to be benign by in silico tools.

*TLR7,* located in a non-pseudoautosomal X chromosome region, has unclear X-inactivation status. Some studies suggest that it is subjected to X-inactivation [56, 57], while others report biallelic expression in immune cells [56, 58]. With the aim of shedding some light into this controversy, we tested the hypothesis of skewed X-inactivation driving severe COVID-19 phenotype in women carrying N215S and D332G variants performing an X-chromosome inactivation assay. Our results did not indicate skewed X-inactivation in these carriers, complicating the interpretation of these variants’ impact. In women, biallelic expression has been linked to enhance TLR7-dependent immune response [56, 58], potentially explaining the protective effect of female sex against severe COVID-19. However, our analysis, performed in DNA from blood cells, could not discard a monoallelic expression defect of TLR7 in pDCs, the primary producers of type I IFNs [22], as they were unavailable for the present study. In addition, other studies hypothesize that heterozygous females may present a dominant-negative effect, in which the TLR7 affected monomer would interfere in the dimerization, thus reducing TLR7 function [28]. Further studies from different approaches are needed to fully elucidate the impact of LOF variants in women.

From our data, it can be observed that three common *TLR7* variants could be potentially implicated in the development of moderate to severe disease presentation: Q11L, c.4-151A>G and c.*881C>G. These findings align with previous publications [30, 32, 33, 59], such as the study by Alseoudy et al. that reported an association between Q11L and COVID-19-related pneumonia [30]. Pre-existing data on the *TLR7* Q11L variant demonstrated reduced in vitro IFN responses following TLR7 agonist administration [60, 61]. A poor IFN response in Q11L carriers was associated with an increased risk of infection and disease progression in other viral infections [62–69]. The intronic variant c.*881C>G has been linked to critical COVID-19 [32, 33], and El-Hefnawy et al. postulated a possible damaging effect resulting from a *TLR7*-driven cytokine storm. Patients harbouring the c.*881C>G variant present *TLR7* mRNA overexpression, which could trigger inflammasome and a dysregulated cytokine storm [32]. Notably, our study is the first that correlates the c.4-151A>G variant with severe COVID-19, whereas it has previously been associated with disease severity and mortality following Crimea-Congo hemorrhagic fever infection [70].

In 2022, gain-of-function (GOF) variants in *TLR7* were first identified as a monogenic cause of systemic lupus erythematosus (SLE) in women [71]. Later, their phenotype description expanded to neuro-inflammatory diseases [53]. Additionally, common *TLR7* variants have also been linked to SLE development [72, 73]. This dual pathogenic role highlights *TLR7*’s central role in type I interferon-mediated innate immune response and inflammation. Exogenous ssRNAs from viruses like SARS-CoV-2 [6, 22] and self-derived ssRNAs from disrupted cells may explain distinct mechanisms underlying two different diseases. Hence, patients with *TLR7* LOF variants exhibit IFN-mediated innate immunodeficiency whereas patients with *TLR7* GOF variants are predisposed to neuroinflammation and/or autoimmune diseases. Table 3 summarizes reported *TLR7* variants and clinical correlations. Recently, LOF variants in *UNC93B1* have been associated with severe COVID-19 susceptibility [35], whereas GOF variants in the same gene have been shown as SLE-causing [74–76]. These findings underscore the relevance of the TLRs-UNC93B1 axis and endosomal trafficking in immunodeficiency and immune dysregulation [42].

**Table 3:** Summary of rare *TLR7* pathogenic variants and clinical phenotype reported in the literature

Gene	HGVSc	HGVSp	Variant	MAF	Effect*	Inheritance	Clinical Phenotype	References
*TLR7*	c.82A>G	p.Arg28Gly	R28G	-	GOF	XLD	Systemic Lupus Erythematous	71
	c.123T>G	p.Asp41Glu	D41E	8.25e-7	LOF^±^	XLR	Severe COVID-19 Susceptibility	37
	c.223A>C	p.Asn75His	N75H	-	LOF	XLR	Severe COVID-19 Susceptibility	22
	c.401T>C	p.Leu134Pro	L134P	-	LOF	XLR	Severe COVID-19 Susceptibility	22
	c.471delC	p.Asn158Thrfs*11	N158 Tfs11*	-	LOF	XLR	Severe COVID-19 Susceptibility	22
	c.644A>G	p.Asn215Ser	N215S	-	LOF	XLR	Severe COVID-19 Susceptibility	21
	c.655G>A	p.Val219Ile	V219I	1.27e-3	Hypomorphic/Neutral	XLR	Severe COVID-19 Susceptibility	22,29
	c.680delT	p.Leu227fs*	L227fs*	-	LOF	XLR	Severe COVID-19 Susceptibility	22
	c.730G>T	p.Asp244Tyr	D244Y	-	LOF	XLR	Severe COVID-19 Susceptibility	22
	c.790T>C	p.Tyr264His	Y264H	-	GOF	XLD	Systemic Lupus Erythematous	71
	c.863C>T	p.Ala288Val	A288V	2.15e-5	Hypomorphic/Neutral	XLR	Severe COVID-19 Susceptibility	22,29
	c.901T>C	p.Ser301Pro	S301P	-	LOF	XLR	Severe COVID-19 Susceptibility	22,29
	c.928T>C	p.Phe310Leu	F310L	-	LOF	XLR	Severe COVID-19 Susceptibility	22
	c.995A>G	p.Asp332Gly	D332G	7.44e-6	Hypomorphic	XLR	Severe COVID-19 Susceptibility	22
	c.1114C>T	p.Leu372Met	L372M	-	Hypomorphic	XLR	Severe COVID-19 Susceptibility	22
	c.1343C>T	p.Ala448Val	A448V	4.16e-3	Hypomorphic/ Neutral	XLR	Severe COVID-19 Susceptibility	22,29
	c.1286_1389dup	p.His464Ilefs*7	H464Ifs*7	-	Not performed	XLR	Post-COVID-19 neurological deterioration	39
	c.1514T>C	p.Ile505Thr	I505T	8.26e-7	LOF	XLR	Severe COVID-19 Susceptibility	22
	c.1520T>C	p.Phe507Ser	F507S	-	GOF	XLD	Systemic Lupus Erythematous/Aicardi-Goutières Syndrome	53
	c.1521T>G	p.Phe507Leu	F507L	-	GOF	XLD	Systemic Lupus Erythematous	53,71
	c.1582C>A	p.Leu528Ile	L528I	-	GOF	XLD	Systemic Lupus Erythematous/Aicardi-Goutières Syndrome	53
	c.1888C>T	p.His630Tyr	H630Y	-	LOF	XLR	Severe COVID-19 Susceptibility	22,29
	c.1970T>C	p.Ile657Thr	I657T	-	LOF	XLR	Severe COVID-19 Susceptibility	22
	c.2010_2011del ;2013_2014insC	p.Phe670Leufs*8	F670Lfs*8	-	LOF	XLR	Severe COVID-19 Susceptibility	22
	c.2050A>T	p.Lys684*	K684*	-	LOF	XLR	Severe COVID-19 Susceptibility	22
	c.2129_2132delAACT	p.Gln710 Argfs*18	Q710Rfs*18	-	LOF	XLR	Severe COVID-19 Susceptibility	12,22
	c.2143C>T	p.Pro715Ser	P715S	-	Hypomorphic	XLR	Severe COVID-19 Susceptibility	22
	c.2342A>T	p.His781Leu	H781L	-	LOF	XLR	Severe COVID-19 Susceptibility	22
	c.2383G>T	p.Val795Phe	V795F	-	LOF	XLR	Severe COVID-19 Susceptibility	12,22
	c.2759G>A	p.Arg920Lys	R920K	1.19e-4	LOF^±^/Neutral	XLR	Severe COVID-19 Susceptibility	22,29,37
	c.2797T>C	p.Trp933Arg	W933R	-	LOF^±^	XLR	Severe COVID-19 Susceptibility	21
	c.2963T>C	p.Leu988Ser	L988S	2.23e-5	LOF	XLR	Severe COVID-19 Susceptibility	22
	c.3094G>A	p.Ala1032Thr	A1032T	5.63e-4	LOF/Neutral	XLR	Severe COVID-19 Susceptibility	22,29

*MAF* minor variant allele frequency according to gnomAD v4; *Based on published functional studies. ± Functional validation was performed only in PBMCs

Our study faces limitations, such as a smaller control sample of SARS-CoV2-infected patients without a healthy volunteer group, and the broad phenotypic variability within the cases. However, its strengths outweigh these constraints. Comprehensive *TLR7* sequencing, along with a detailed analysis of both common and rare variants, provide valuable insights. The study’s reliability is further reinforced by precise clinical characterization and the exclusion of AAN-IFN-I. Although SARS-CoV-2 serologies were not performed in all patients to rule out prior asymptomatic infections or cross-immunity, patient classification as naïve or primo-infected was conducted thoroughly and consistently based on medical records.

## Conclusions

In summary, our study establishes a compelling link between *TLR7* LOF variants in men and increased susceptibility to severe COVID-19, exemplified by the N215S variant, which completely abolishes signal transduction upon stimulation. We validate the luciferase reporter assay as a robust and reproducible platform for characterizing the functional impact of *TLR7* variants. Additionally, we identify associations between several common *TLR7* variants and the development of moderate to severe COVID-19 presentations. Collectively, our findings position *TLR7* as a critical genetic determinant of disease severity and a strong candidate for further investigation in the context of RNA-virus-associated pathologies. These insights have broad implications for understanding individual variability in disease outcomes and may inform future strategies for genetic screening, risk assessment, and the development of targeted therapeutic interventions.

## Supplementary Information

Below is the link to the electronic supplementary material.

ESM 1 (PNG 420 KB)

Supplementary file1 (TIFF 34881 KB)

ESM 2 (PNG 155 KB)

Supplementary file2 (TIF 45718 KB)

Supplementary file3 (XLSX 70 KB)

## Data Availability

No datasets were generated or analysed during the current study.
