# Supplementary figures and images for: From Rare to Common: Genetic Insights into TLR7 Variants in a Multicentric Spanish Study on COVID-19 Severity
Source: J Clin Immunol. 2025 May 27;45(1):100. doi: 10.1007/s10875-025-01892-0 (PMC12116960; doi:10.1007/s10875-025-01892-0)

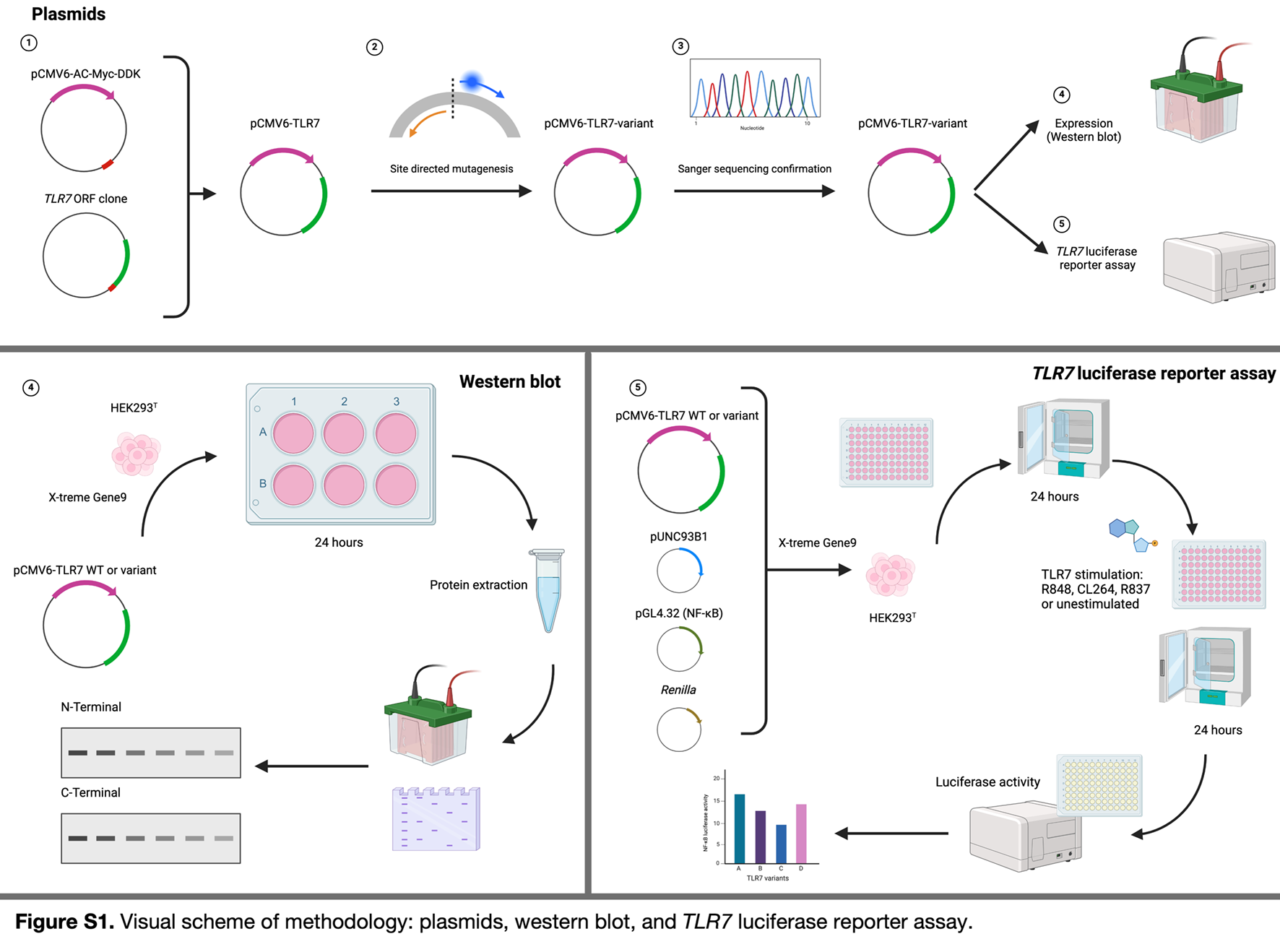

Supplement: Supplementary file 1 — (PNG 420 KB) [file 10875_2025_1892_Fig3_ESM.png]

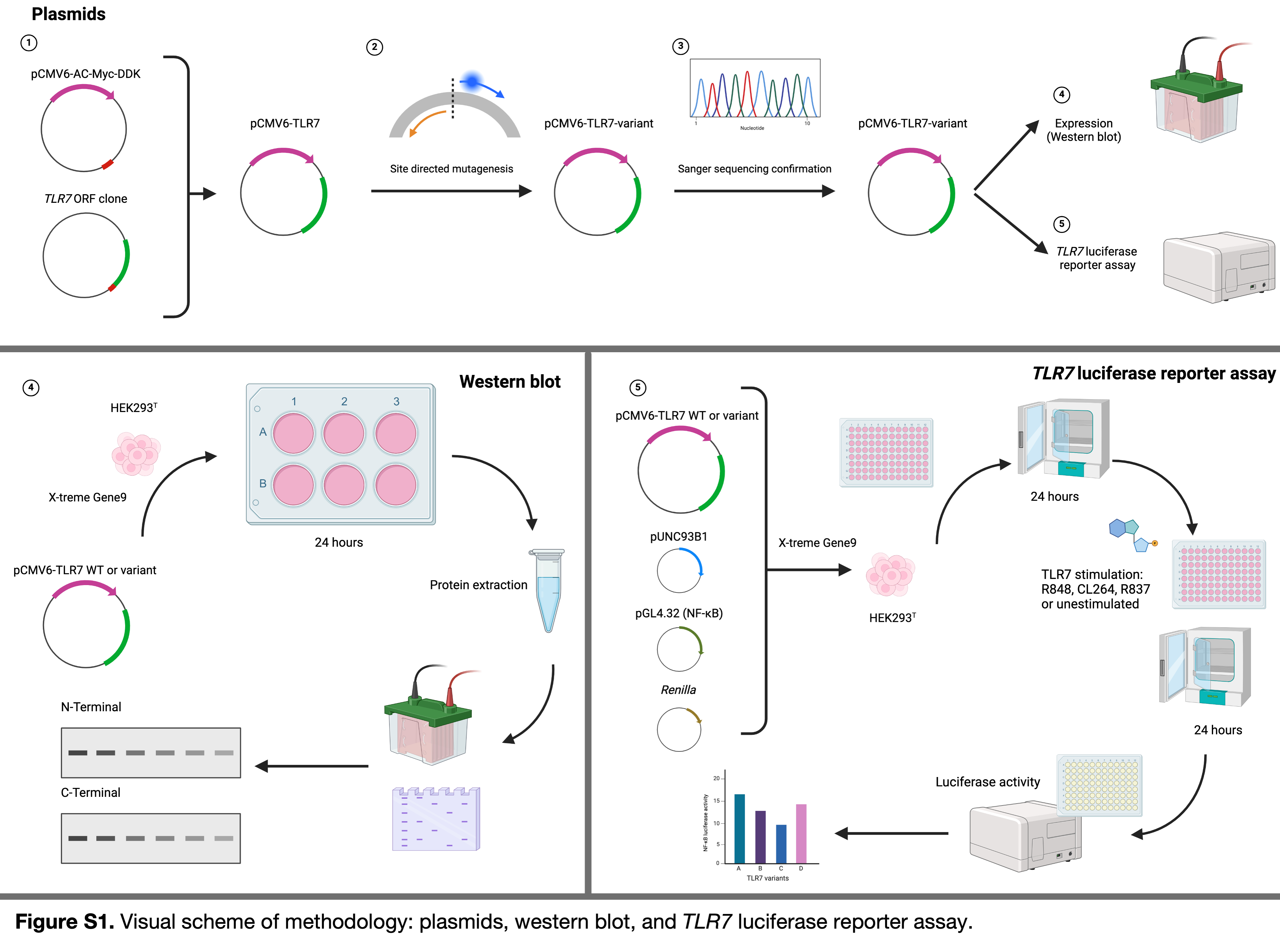

Supplement: Supplementary file 2 — Supplementary file1 (TIFF 34881 KB) [file 10875_2025_1892_MOESM1_ESM.tiff]

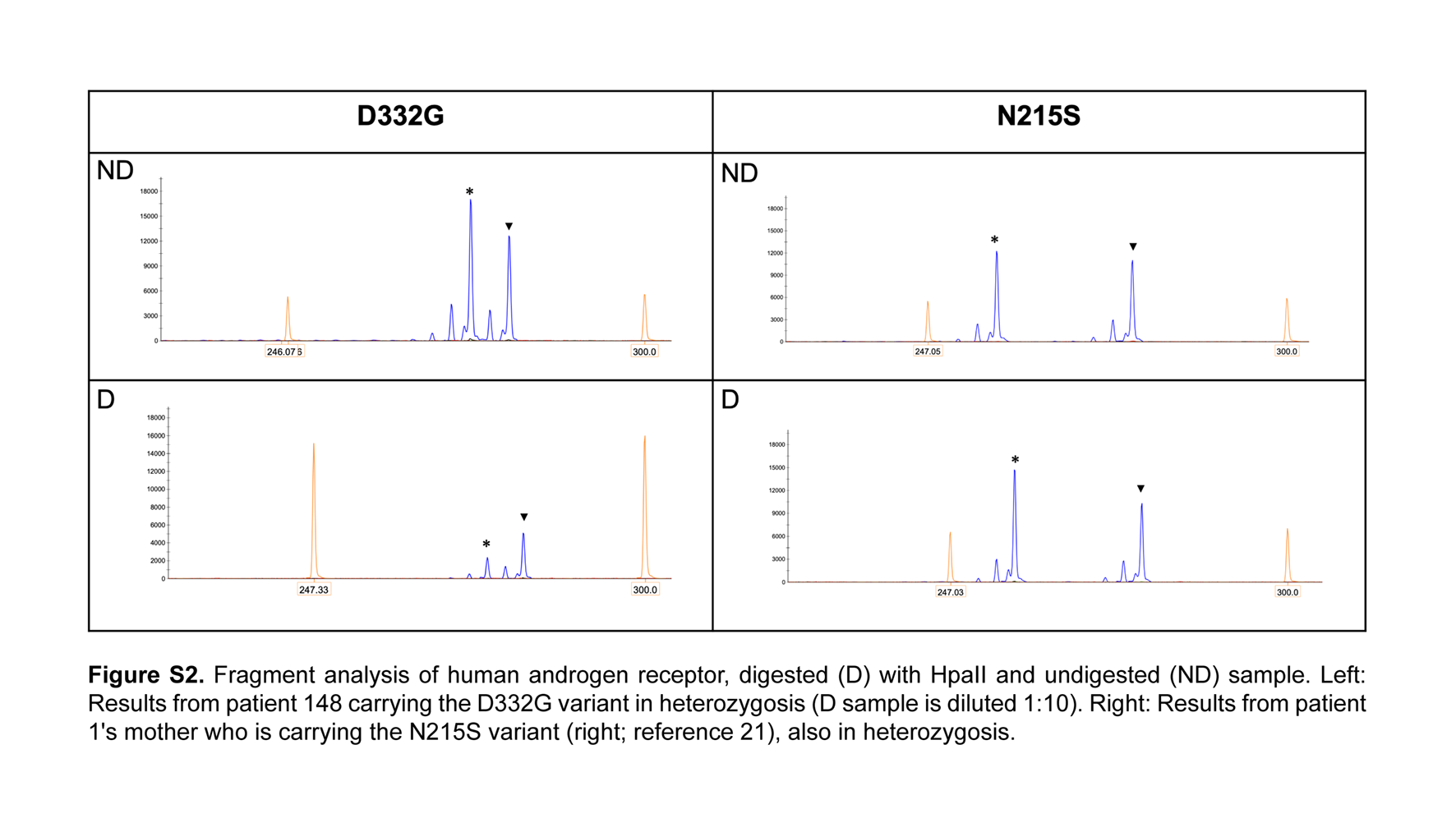

Supplement: Supplementary file 3 — (PNG 155 KB) [file 10875_2025_1892_Fig4_ESM.png]

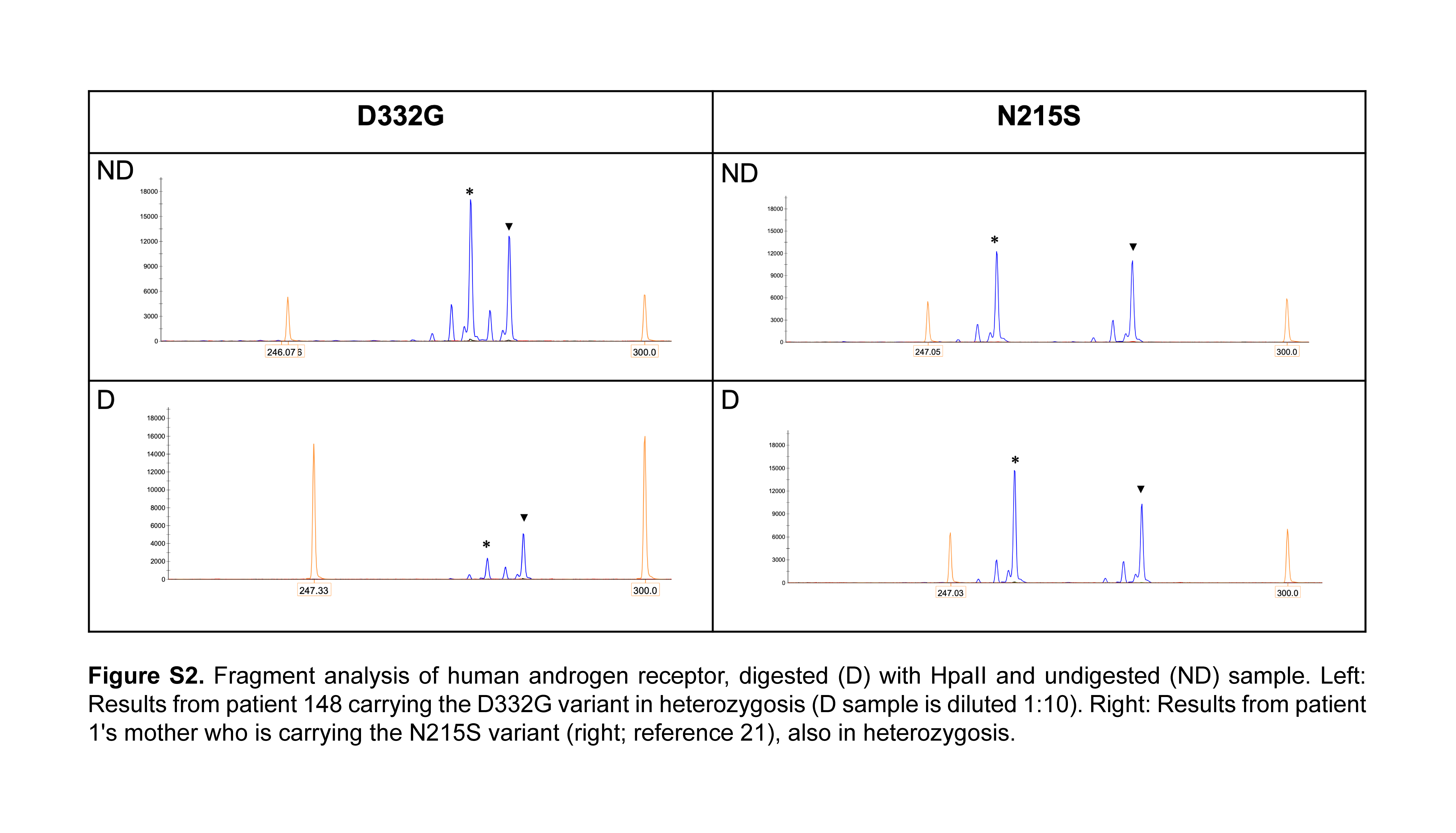

Supplement: Supplementary file 4 — Supplementary file2 (TIF 45718 KB) [file 10875_2025_1892_MOESM2_ESM.tif]
